# Early identification and treatment for peripheral arterial disease in patients with ischemic cerebrovascular disease

**DOI:** 10.1186/s40001-023-01050-5

**Published:** 2023-02-23

**Authors:** Lu-guang Li, Xin Ma

**Affiliations:** 1 Department of Neurology, Xuanwu Hospital, Capital Medical University, No. 45 Changchun Street, Xicheng District, Beijing, 100053 China; 2 National Clinical Research Center for Geriatric Disorders, Beijing, China; 3 Clinical Center for Cardio-Cerebrovascular Disease of Capital Medical University, Beijing, China

**Keywords:** Peripheral arterial disease, Ischemic cerebrovascular disease, Coexistence, Diagnosis, Therapy

## Abstract

Ischemic cerebrovascular disease (ICVD) is a major cause of mortality and disability worldwide and is often caused by atherosclerosis. As a systemic disease, atherosclerosis usually affects multiple vascular beds, mainly including cerebral, coronary, and peripheral arteries. Therefore, ICVD is easily complicated by lower-extremity peripheral arterial disease (PAD). ICVD patients with PAD have more serious symptoms and a worse prognosis, however, neurologists might neglect the evaluation and management of the coexistent PAD, and there is still a lack of consensuses about the diagnosis and treatment for such patients. By summarizing relevant research progresses, this review showed that duplex ultrasound had more advantages in the early screening and evaluation of PAD in ICVD patients among multiple methods to diagnose PAD. Furthermore, the current evidence seems to support that single-drug antiplatelet can be used as the basic treatment, and new antithrombotic strategies, such as ticagrelor only or aspirin combined with low-dose rivaroxaban are expected to further reduce the incidence of stroke for ICVD patients with PAD. More effective treatments would be explored by large-scale trials to guide the clinical management to prevent secondary stroke for such patients.

## Background

Ischemic cerebrovascular disease (ICVD) does great harm to human health with a mortality rate of 20% and a disability rate of 55% [1, 2], and is often caused by cerebral arteries atherosclerosis (AS). As a systemic disease, AS commonly affects multiple vascular beds, mainly including cerebral, coronary, and peripheral arteries [3]. Therefore, ICVD is easily complicated by lower-extremity peripheral arterial disease (PAD) [4]. PAD usually refers to atherosclerotic lesions involving iliac, femoral, and lower-extremity arteries [5]. It is estimated that approximately 200 million people are suffering from PAD around the world, and its prevalence is still rising [6]. Clinical studies have clarified that about one-fifth of ICVD patients have PAD [7], and ICVD patients with PAD have more serious symptoms and worse prognosis [3, 8, 9]. There will be problems in the systematic diagnosis and treatment of ICVD patients with PAD. However, neurologists tend to only focus on the treatment of ICVD and might neglect the coexisting PAD. Besides, although PAD diagnosis and treatment guidelines have been published internationally, there is still a lack of consensuses in the systematic management of ICVD patients with PAD. The most reasonable method for early screening of PAD among ICVD patients is not clear, and the optimal antithrombotic strategy remains controversial. We reviewed and analyzed current literature and discussed the relevant diagnostic and therapeutic regimens for such patients to provide some reference for clinical practice.

## Impact of the coexisting PAD on the severity and prognosis of ICVD patients

It seems that PAD is increasingly common [6]. Large-scale studies among general population showed that the prevalence of PAD is about 2.9–4.1%, which is not significantly lower than the 3.9–6.7% of ICVD [10–12]. The coexistence rate of PAD and ICVD is also relatively higher because atherosclerotic diseases share similar risk factors, such as hypertension, diabetes, hyperlipidemia, and smoking [13]. The current research suggested that about 12.2–18% of ICVD patients were complicated by PAD [4, 7, 14], and about 20–23.3% of PAD patients also had ICVD [10, 15]. The prevalence of PAD in ICVD patients is much greater than that in the general population, which needs full attention because such patients have a poor prognosis. The REduction of Atherosclerosis for Continued Health (REACH) registry was a multicenter cohort study involving 67,888 patients, and all patients had stable vascular diseases (coronary artery disease (CAD), ischemic stroke (IS), transient ischemic attack (TIA), or PAD), or multiple vascular risk factors. The results showed when patients had polyvascular diseases, including ICVD coexisting with PAD, the 3-year rates of vascular death increased by 4%, and the primary endpoints (myocardial infarction (MI), stroke, or vascular death) increased by 7% when compared with single-bed disease [7]. ICVD patients with PAD having a higher incidence of vascular events could be explained by the severity of cerebral artery AS. A cross-sectional study included 106 IS patients found that the number of patients with moderate (50–69%) and severe (70–100%) intracranial artery stenosis (ICAS) in PAD group was significantly higher than that in PAD free group (35.7% vs 4.3%, *P* < 0.01), an only PAD was independently associated with increasing ICAS grades (OR: 4.32, 95% CI 1.35–13.80; *P* = 0.013) [16]. ICAS is a major cause of stroke occurrence and recurrence [17], so patients with more severe ICAS may have a higher probability of recurrent stroke. A meta-analysis addressed 11 studies with a total of 5374 IS or TIA patients, and the results showed that PAD was associated with an increased risk of stroke recurrence (HR: 1.70, 95% CI 1.10–2.64) and vascular events or vascular death (HR: 2.22, 95% CI 1.67–2.94) [18]. In addition, ICVD was positively correlated with the severity of PAD. The Examining Use of Ticagrelor in Peripheral Artery Disease (EUCLID) trial included 13,885 symptomatic PAD patients, and the ankle brachial index (ABI) was measured in all patients. ABI measurement is one of the screening methods for PAD, ABI less than 0.9 can establish the diagnosis and a lower ABI often means a more serious PAD [19]. It was found that patients with severe PAD (baseline ABI < 0.60, prior amputation, or critical limb ischemia) had a higher incidence of stroke than patients with ABI < 0.9 (*P* = 0.005), and lower ABI was independently associated with the occurrence of all-cause stroke [20]. The conclusion may be explained by the risk factors leading to the PAD progression, such as age, smoking, diabetes, and dyslipidemia overlap with the risk factors of ICVD [21], but research is still needed to further explore the relevant mechanisms.

Overall, about one-fifth of ICVD patients suffer from PAD, and ICVD patients with PAD have more serious symptoms and a worse prognosis. At present, PAD has been regarded as a risk-enhancing factor in the progression of atherosclerotic diseases [22], and neurologists should attach importance to the early screening and evaluation of PAD in ICVD patients. However, the most appropriate screening method is still controversial.

## Early screening methods for PAD in ICVD patients

When ICVD patients come to a neurologist, some clinical manifestations of PAD could be easily neglected because they are not related to their specialty. Neurologists should be aware of the typical symptoms and physical examination manifestations of PAD, such as intermittent limb pain, claudication, chronic rest pain, or a weak lower-extremity arterial pulsation. ICVD Patients with such manifestations should be actively carried out relevant examinations to clarify the diagnosis of PAD [23].

There are multiple methods to diagnose PAD, including ABI, duplex ultrasound (DUS), computed tomography angiography (CTA), magnetic resonance angiography (MRA), and digital subtraction angiography (DSA). Among them, ABI and DUS are two first-line methods for noninvasive, cost-effective, and quickly screening for PAD in large-scale populations [24, 25]. Although CTA and MRA are indicated for further determining the anatomic location and the severity of stenosis when revascularization is considered [19], they lack functional and hemodynamic data, contrast agents of CTA present potential risks, and the costs are relatively higher, hence, they are not suitable for PAD screening [26]. Although DSA remains the gold standard for PAD diagnosis, it is no longer regarded as a first-line method to diagnose PAD for its invasiveness and risk of complications [19].

ABI is the ratio between systolic blood pressures (SBP) measured at the ankle and brachial artery of a patient in the supine position [19]. ABI less than 0.9 can establish the diagnosis of PAD [19]. It can preliminarily evaluate the ischemia of peripheral arteries to reflect its perfusion status as an efficient, simple, and most economic screening test [27]. The main drawback is that ABI cannot accurately locate the vascular lesion and is unable to determine the morphological changes in vessels. Its sensitivity will also be significantly reduced when patients suffer from diseases involving small vessels, such as diabetes because such diseases are more likely to co-exist with medial arterial calcification which can prevent compression and pressure measurement [28, 29]. Therefore, a normal ABI does not exclude the presence of PAD [27]. Another study reviewed the efficiency of ABI in the diagnosis of PAD and found that its sensitivity was between 61–96% and the specificity ranged from 56 to 90% [30].

DUS has been increasingly used in the diagnosis of PAD recently because of its noninvasive, high detection rate, and repeatability. When compared with ABI, DUS can further determine vascular anatomy, hemodynamics, lesion morphology, and the severity of stenosis [31], and the results in diagnosing PAD are not easily affected by blood pressure (BP) fluctuation, body position, or latent small vessel disease [26]. Retrospective studies compared the efficacy of ABI and DUS in the diagnosis of PAD and found that in patients with lower-extremity artery stenosis > 50% on DUS examination, about 40% had normal/inconclusive resting ABI [32, 33], which means that ABI has limited sensitivity for the detection of PAD compared with DUS. Some reviews concluded that the sensitivity and specificity of DUS were 79.7–97% and 88.5–99% respectively, which were significantly higher than the 70% and 90% of ABI [30, 34]. From the perspective of treatment, extended AS and an increased atherosclerotic burden in PAD is one of the reasons that pharmacological prevention of vascular events is less effective [35]. DUS could early identify the extension of AS and therefore adopt a more targeted therapeutic regime. There is no unified DUS diagnostic standard for PAD so far. Most studies measure the peak systolic velocity (PSV) of lower-extremity artery segments through the doppler spectrum, and PAD can be diagnosed when the PSV at the stenosis is ≥ 200 cm/s or the PSV ratio ≥ 2 at the stenosis to the proximal [36, 37]. A reduction of arterial lumen diameter ≥ 50% measured by B-mode ultrasound could also be taken as a diagnostic standard [30].

At present, ABI is still the main screening method for PAD among large sample studies internationally [23], which is also in line with current US and European guidelines [19]. However, most PAD in ICVD patients is asymptomatic lower-extremity arterial stenosis with an inferior detection rate by ABI measurement. By contrast, DUS can directly identify arterial intimal thickening and lumen stenosis and has a higher sensitivity for asymptomatic patients [38]. Therefore, DUS screening may be cost-effective in the early identification and intervention of ICVD patients with PAD because of its higher sensitivity and specificity and the possibility of guiding treatment. The latest Chinese guideline also regards DUS as a first-line method for PAD screening [39]. DUS could be more widely used for detecting PAD in ICVD patients in future, and more research should be carried out to compare the effectiveness of DUS and ABI in screening PAD among ICVD patients to clarify the best diagnostic scheme.

## Treatment strategies for ICVD patients with PAD

### Management of risk factors

Traditional risk factors of PAD are similar to those of ICVD, mainly including hypertension, diabetes, chronic kidney disease, hyperlipidemia, and smoking [13]. However, although the pathophysiology underlying remains unclear, latest research has emphasized a differential pattern between some risk factors and AS in different vascular beds [22]. Such discrepancy might lead to differences in BP and cholesterol management strategies between ICVD patients with and without PAD.

BP has an independent and continuous relationship with the incidence of vascular events, and hypertension is a major risk factor for ICVD and PAD [40]. A BP goal of < 130/80 mmHg is recommended for most neurologically stable ICVD patients to reduce cardiovascular events and stroke recurrence [41]. For PAD patients, it is recommended to control BP at < 140/90 mmHg to reduce vascular events [19]. Although intensive control of SBP to 90–120 mmHg for ICVD patients does not increase stroke recurrence [41], reducing SBP to < 110–120 mmHg can lead to an increase of cardiovascular events in PAD patients [42]. Therefore, an SBP > 130 mmHg or < 110 mmHg may not be appropriate for most ICVD patients with PAD. To be emphasized, the BP goal of < 130/80 mmHg may not be applicable to patients with acute IS or severe large arterial stenosis [43], and the optimal BP target for ICVD patients with PAD still needs to be explored. As for drug selection in ICVD patients with hypertension, the latest guideline suggested that angiotensin-converting enzyme inhibitors (ACEIs), angiotensin receptor blockers (ARBs), or diuretics had demonstrated benefit in randomized controlled trials (RCTs) for secondary stroke prevention (IA) [41]. In PAD patients with hypertension, guidelines recommended that ACEIs or ARBs should be considered as first-line therapy because they are associated with decreased cardiovascular events (IIB) [24]. Hence, ACEIs or ARBs seem to be preferred for antihypertension in ICVD patients with PAD. However, there is still a lack of evidence from large-scale studies on the optimal BP target and medication management strategies for such patients.

Cholesterol management is crucial for both ICVD and PAD patients. Serum low-density lipoprotein cholesterol (LDL-C) should be reduced to < 1.8 mmol/l (< 70 mg/dl) for all PAD or ICVD patients according to current guidelines [24, 41]. Several large RCTs including PAD subjects demonstrated that a further reduction of LDL-C based on the high-intensity statin therapy could significantly reduce cardiovascular or cerebrovascular events [44]. Proprotein convertase subtilisin/Kexin type 9 (PCSK9) inhibitors can further reduce LDL-C with a safety profile and are associated with lower atherosclerotic vascular events in PAD patients [45]. An RCT involving 3642 PAD patients showed that the addition of PCSK9 inhibitors to high-intensity statin therapy reduced the relative risk of stroke by 22% (*P* < 0.01) [46]. Meanwhile, a recent meta-analysis showed that intensive cholesterol-lowering therapy significantly reduced major adverse vascular events in patients with multivascular diseases compared with single vascular disease (6.5% vs 1.8%) [47]. Based on the research above and other evidence, the latest guideline for secondary stroke prevention for IS patients with symptomatic PAD, suggested that a PCSK9 inhibitor should be added if LDL-C is > 1.8 mmol/l when treated with high-intensity statin combined with ezetimibe [41]. Ezetimibe was recommended to be used before the addition of PCSK9 inhibitor to further lower LDL-C for such patients when LDL-C is > 1.8 mmol/l [41]. However, the evidence level of this recommendation is relatively low (II B-NR), and it is unclear whether further reducing LDL-C in ICVD patients with PAD can better for preventing cerebrovascular events, optimal cholesterol management strategies for such patients still need further exploration.

#### Antithrombotic therapy

Increasingly evidence shows that the enhancement of platelet activity and atherosclerotic thrombosis have a significant effect on the PAD formation. Antithrombotic therapy has shown great benefits and plays an increasingly important role in PAD management [48]. However, the plaque composition and arterial occlusion in the lower extremities are not equivalent to those of the cerebral circulation [35]. Most lesions in peripheral arteries are predominantly fibroproliferative with a relatively small amount of lipids and inflammatory cells, which probably makes them more stable and less vulnerable to standard long-term single antiplatelet therapy (SAPT) [35]. Therefore, the antithrombotic strategy in PAD patients is still controversial, and the treatment schemes for symptomatic and asymptomatic patients are also different.

Whether antithrombotic therapy is needed in asymptomatic PAD patients has not yet reached a consensus, and such patients are often neglected in clinical practice [25, 49]. One RCT included 3350 asymptomatic PAD patients with ABI < 0.99, and results showed that aspirin did not significantly reduce the incidence of cardiovascular or cerebrovascular events compared with placebo [50]. Another study compared aspirin against placebo on 1,276 asymptomatic PAD patients with ABI < 1.0 and showed that there was no significant difference in the primary endpoint (stroke or MI or amputation) (HR 0.98, 95% CI 0.76–1.26) [51]. However, both studies did not use standard ABI (< 0.9) to diagnose PAD, so a part of the normal population may be mixed [52]. Because most ICVD patients have already taken aspirin SAPT for secondary stroke prevention, there is no evidence to support the additional use of other antiplatelet drugs in ICVD patients with asymptomatic PAD. Aspirin SAPT seems to be reasonable, and more clinical studies are needed to confirm this conclusion.

Previous studies suggested that the antithrombotic drug selection of symptomatic PAD patients includes antiplatelets and anticoagulants [53]. Antiplatelet drugs were shown to delay the progression of lower-extremity symptoms because PAD is related to abnormal platelet activity, excessive aggregation, and enhanced adhesion [54]. Antiplatelet drugs also present a protective effect on cardiovascular and cerebrovascular events for both ICVD and symptomatic PAD patients [55]. The recent guidelines suggest that long-term SAPT should be used in symptomatic PAD patients to prevent stroke (IA), and antiplatelet therapy could also reduce limb ischemia in such patients [19]. In most cases, aspirin is the first choice for long-term SAPT in ICVD patients [41], but the optimum selection for symptomatic PAD patients remains controversial. Previous studies found no difference in IS events between clopidogrel and aspirin SAPT in PAD patients, but total events (IS, MI, or vascular death) in the clopidogrel group was lower (3.71% VS 4.86%, *P* < 0.01) [56]. Ticagrelor SAPT was associated with a lower adjusted rate of ischemic (HR 0.78; 95% CI 0.62–0.98; *P* = 0.032) and all-cause stroke (HR 0.80; 95% CI 0.64–0.99; *P* = 0.038) than clopidogrel, although the primary endpoint (cardiovascular death, MI, or IS) was not significantly different (10.6% versus 10.8%) [57]. Hence, ticagrelor SAPT seems to be more effective than aspirin in the prevention of ICVD in patients with symptomatic PAD. However, no study directly compared the effects of ticagrelor and aspirin in PAD patients, and the role of ticagrelor for secondary stroke prevention in ICVD patients is not well established [41], so further explorations are still needed.

Studies have shown that the short-term application of dual antiplatelet therapy (DAPT) can further reduce stroke recurrence compared with SAPT in mild IS or TIA patients. The current stroke prevention guideline recommended starting short-term DAPT for such patients as soon as possible (IA) [41]. However, DAPT shows no obvious advantages over SAPT when long-term used in ICVD patients [58]. For symptomatic PAD patients, two trials showed that there was no significant difference between aspirin combined with clopidogrel and aspirin alone to prevent stroke or other vascular events [59, 60]. The addition of ticagrelor to aspirin resulted in a significant reduction in cardiovascular mortality for symptomatic PAD patients (HR 0.47; 95% CI 0.25–0.86; *P* = 0.014) but did not reduce the risk of stroke (HR 0.49; 95% CI 0.21–1.14; *P* = 0.097) [61]. Hence, the long-term application of DPAT to prevent stroke still lacks evidence, and it seems preferable to use aspirin or clopidogrel SAPT to treat and prevent cerebrovascular events in ICVD patients with symptomatic PAD. Large-scale clinical trials should be conducted for such patients to determine the most reasonable antiplatelet treatment scheme.

Cilostazol is another antiplatelet option for ICVD patients. Studies on ICVD patients have shown that it might be superior to aspirin for secondary stroke prevention with fewer bleeding episodes [62], and adding cilostazol to aspirin or clopidogrel could further reduce stroke recurrence in high-risk ICVD patients [63]. Meanwhile, cilostazol has been used to improve symptoms in PAD patients for decades because it can dilate vessels and enhance blood supply. Its effectiveness in the treatment of intermittent claudication has been fully confirmed and written into guidelines [25]. However, there is no study comparing the therapeutic effects of cilostazol and other antiplatelet drugs on PAD patients, so it has not been widely accepted as an antiplatelet option to prevent stroke in patients with PAD, and further comparative RCTs may be needed to provide evidence for expanding its indications.

Single-drug anticoagulants were not recommended in guidelines for symptomatic PAD patients because previous trials have shown that the addition of warfarin or edoxaban could not reduce stroke compared with aspirin [64]. However, the Cardiovascular Outcomes for People Using Anticoagulation Strategies (COMPASS) trial recently revealed that aspirin combined with low-dose rivaroxaban has a preferable therapeutic effect on symptomatic PAD patients, which provides a new idea for the antithrombotic treatment of ICVD patients with PAD. Rivaroxaban is a highly selective direct FXa inhibitor. In addition to inhibiting thrombin formation and thrombus development [65], it can effectively inhibit platelet aggregation induced by tissue factor, which may be helpful to prevent arterial thrombosis [66]. COMPASS trial included 27,395 randomly assigned participants with stable CAD or PAD, and patients were randomly arranged to receive aspirin (100 mg once daily) or rivaroxaban (5 mg twice daily) or low-dose rivaroxaban (2.5 mg twice daily) with aspirin (100 mg once daily). The results showed that rivaroxaban 2.5 mg twice daily combined with aspirin significantly reduced strokes when compared with aspirin only (HR 0.58; 95% CI 0.44–0.76; *P* < 0.0001) [67]. Subgroup analysis showed that low-dose rivaroxaban plus aspirin reduced primary outcomes (cardiovascular death, MI, and stroke) compared with aspirin alone in patients with symptomatic PAD (HR 0.72, 95% CI 0.57–0.90, *P* = 0.0047), and fatal or critical organ bleeding did not increase [68], so the efficacy and safety of aspirin combined with low-dose rivaroxaban have been confirmed in symptomatic PAD patients. However, there is no study to explore the safety and efficacy of rivaroxaban in ICVD patients, and the addition of rivaroxaban to aspirin might increase the incidence of major bleeding. The efficacy of rivaroxaban combined with aspirin in ICVD patients with PAD needs to be further confirmed.

At present, the antithrombotic drug selection of most ICVD patients is long-term aspirin or clopidogrel SAPT, which is also applicable to PAD patients, so it can be used as the basic strategy for ICVD patients with PAD. Ticagrelor SAPT or aspirin combined with low-dose rivaroxaban might be suitable for symptomatic PAD patients, however, reliable evidence for these novel antithrombotic therapies seems insufficient. In addition, more effective treatments would be explored by large-scale trials to guide the clinical management to prevent secondary stroke for ICVD patients with PAD and other patients with polyvascular atherosclerotic diseases.

## Conclusion

About one-fifth of ICVD patients suffer from PAD, and ICVD patients with PAD have more serious symptoms and a worse prognosis. DUS provides more valuable information with higher sensitivity and specificity for screening PAD in ICVD patients than ABI measurement. Current evidence seems to support that single-drug antiplatelet can be used as the basic treatment, and new antithrombotic strategies such as ticagrelor only or aspirin combined with low-dose rivaroxaban are expected to further reduce the incidence of stroke for ICVD patients with PAD. More effective treatments should be explored and confirmed by large-scale trials to guide more intensive management for such patients.

## Data Availability

Data sharing is not applicable to this article as no datasets were generated or analysed during the current study.

## Abbreviations

ACEIs: Angiotensin-converting enzyme inhibitors
ABI: Ankle brachial index
ARBs: Angiotensin receptor blockers
AS: Atherosclerosis
BP: Blood pressure
CAD: Coronary artery disease
COMPASS: Cardiovascular outcomes for people using anticoagulation strategies
CI: Confidence interval
CTA: Computed tomography angiography
DAPT: Dual antiplatelet therapy
DUS: Duplex ultrasound
DSA: Digital subtraction angiography
EUCILD: Examining Use of Ticagrelor in Peripheral Artery Disease
HR: Hazard ratio
ICAS: Intracranial artery stenosis
ICVD: Ischemic cerebrovascular disease
IS: Ischemic stroke
LDL-C: Low-density lipoprotein cholesterol
MI: Myocardial infarction
MRA: Magnetic resonance angiography
OR: Odds ratio
PAD: Peripheral arterial disease
PCSK9: Proprotein convertase subtilisin/Kexin type 9
PSA: Peak systolic velocity
REACH: REduction of Atherosclerosis for Continued Health
SAPT: Single antiplatelet therapy
SBP: Systolic blood pressure
TIA: Transient ischemic attack
